# Impact of DHA from Algal Oil on the Breast Milk DHA Levels of Lactating Women: A Randomized Controlled Trial in China

**DOI:** 10.3390/nu14163410

**Published:** 2022-08-19

**Authors:** Yue Yang, Genyuan Li, Fang Li, Fangping Xu, Ping Hu, Zhencheng Xie, Xiaolong Lu, Ye Ding, Zhixu Wang

**Affiliations:** 1 Department of School Health, Nanjing Municipal Center for Disease Control and Prevention Affiliated to Nanjing Medical University, Nanjing 210003, China; 2 Department of Maternal, Child and Adolescent Health, School of Public Health, Nanjing Medical University, Nanjing 211166, China

**Keywords:** lactating women, DHA, breast milk, intervention

## Abstract

Population research on the intervention of docosahexaenoic acid (DHA) supplementation in lactating women is in its infancy in China. This study investigated the effect of DHA supplementation on DHA concentrations in the breast milk of lactating women, and the intervention effect, with respect to different dietary patterns. In this trial, 160 healthy lactating women in Nanjing (30–50 days postpartum) were recruited and randomly divided into control (one placebo capsule of similar appearance per day) and supplement (one capsule with 200 mg of DHA from algal oil per day) groups for 8 weeks. Before and after the intervention, all subjects were asked to maintain basic information, maternal anthropometric parameters, breast milk (10–15 mL) sample collection, and a dietary survey using a food frequency questionnaire. The concentrations of DHA and other fatty acids in breast milk were detected using capillary gas chromatography. This study was completed by 137 subjects, with 60 in the control group and 77 in the supplement group. Compared with the DHA concentrations in the breast milk at enrollment, the absolute concentrations of the control group showed a significant decrease at the end of the trial (*p* = 0.037). In addition, after intervention, the absolute and relative DHA concentrations in the supplement group (10.07 mg/100 mL and 0.40%, respectively) were higher than those in the control group (7.57 mg/100 mL and 0.28%, respectively), being statistically significant (*p* = 0.012 and *p* = 0.001). Furthermore, the maternal diet in the supplement group was divided into four dietary patterns. Pattern 1 mainly included fruits and livestock meat. Pattern 2 was dominated by milk and its products, eggs, fish, shrimp and shellfish, and soybeans and its products. Pattern 3 chiefly comprised cereal and beans other than soybeans, potatoes, and nuts. Pattern 4 was high in poultry meat and low in cooking oils. The change in the absolute concentration of DHA in Pattern 3 was lower than that in other patterns (*p* < 0.05). In conclusion, DHA supplementation in lactating mothers increased breast milk DHA concentrations. The dietary pattern mainly characterized by cereal and beans other than soybeans, potatoes, and nuts may contribute to the poor intervention effect.

## 1. Introduction

Breast milk is the optimal natural food for infants [1]. Besides persistent breast milk feeding, the quality and quantity of breast milk also require immense attention. Docosahexaenoic acid (DHA) is an *n*-3 long-chain polyunsaturated fatty acid (PUFA). During pregnancy and infancy, DHA is rapidly enriched in the brain, retina, and other neural tissues of the fetal body, thus facilitating brain development in early life and promoting the formation and functional maintenance of the nervous system. Indeed, DHA plays a key role in cognitive functioning, visual development, and the immune functioning of infants [2,3,4]. Although DHA is a nonessential fatty acid and can be synthesized by α-linolenic acid (ALA) in vivo, the infant’s development is not complete; the desaturase activity is low, which weakens the DHA synthesis ability [5]. Therefore, breast milk is an important source of DHA for exclusively breast-fed infants.

Studies have demonstrated that the DHA content in breast milk is affected by geographical location and dietary habits. Women in coastal or freshwater lake areas have higher DHA content in their breast milk, which is related to their intake of higher levels of DHA-rich marine/aquatic products. However, the intake of DHA in the diets of women in inland areas is relatively low, which affects the DHA levels in their breast milk [6]. Hence, DHA in breast milk is insufficient to meet the needs of infants. In this case, DHA supplementation needs to be considered. To date, intervention studies on lactating women taking DHA supplementation have been reported in the United States and Germany, but the reference data are limited [7,8]. In China, few studies have been conducted on DHA supplementation and most of them are focused on pregnancy intervention or lactation intervention based on animal experiments [9,10]. Indeed, population research on the intervention of DHA supplementation in lactating women is in its infancy.

Over the past decades, the dietary structures of Chinese people have changed immensely owing to rapid economic development [11]. As previously mentioned, the dietary DHA statuses of lactating women may affect the DHA levels in breast milk. Practically, however, the food consumed by lactating women is not just DHA, a kind of nutrient or marine/aquatic products, but a composite diet of various nutrients and food types. It is worth examining whether different dietary conditions of lactating women are likely to impact the DHA content in breast milk after the intervention of DHA supplementation. The findings can aid in ensuring the adequate nutritional status of DHA in this group.

Dietary patterns refer to the quantities of various foods and their proportions in the diet, which can comprehensively reflect the dietary structures of lactating women and the combined effect between food and nutrients [12]. Currently, dietary patterns have attracted attention in the study of diets and health outcomes [13,14]. If the long-term dietary intake statuses of lactating women are collected via food frequency questionnaires (FFQs) and the dietary intake characteristics of the population are divided into several patterns, the impacts of certain patterns on the DHA content in breast milk after the intervention of DHA supplementation can be discussed at the macro level.

In summary, this study aims to clarify the intervention effects of DHA supplementation on the breast milk DHA levels of lactating women in China. The study further explores the intervention effects under different dietary patterns in combination with the dietary statuses of lactating women. Thus, accurate theoretical guidance for the dietary intake of lactating women and the use of nutrient supplementation can be provided. Furthermore, the findings can aid nutritionists in formulating appropriate diets for lactating women.

## 2. Materials and Methods

### 2.1. Subjects

From November 2018 to September 2019, healthy lactating women at 30–50 days postpartum were randomly recruited from the Maternal and Child Health and Family Planning Service Centre of Jiangning District in Nanjing, Jiangsu Province.

Inclusion criteria: healthy lactating women aged 20–40 years who were exclusively breastfeeding for 30–50 days postpartum, had singleton pregnancies, full-term deliveries (≥37 gestation weeks), with no disease, and infants with birth weights ≥ 2500 g.

Exclusion criteria: lactating women with any infectious disease, malignant consumptive disease, malnutrition, or mental disease, those with postpartum emotional distress who did not cooperate well, or those who had been diagnosed with any breast disease and pregnancy complication. Mothers with a history of alcohol consumption and smoking habits during pregnancy or lactation, who were vegetarians, who took fatty acid supplementation during lactation, who recently used hormones and antibiotics, or who participated in any other research on nutrition or drug intervention during the past 30 days.

According to the principle of informed consent, all subjects provided oral and written informed consent; the study was approved by the Ethics Committee of Nanjing Medical University and registered at http://www.chictr.org.cn/index.aspx (ChiCTR 1800020179) (accessed on 19 December 2018).

### 2.2. Study Design

#### 2.2.1. Pattern and Content of the Intervention

The supplement group took one algal DHA oil capsule (each containing 200 mg of DHA sourced from Wyeth) daily, from the next day after enrollment, and took it continuously for 8 weeks until the follow-up visit. The control group took one placebo capsule of a similar appearance.

#### 2.2.2. Basic Information Collection

Each subject answered a standardized questionnaire in a face-to-face interview on sociodemographic characteristics and basic information. Maternal information, such as age, height, pre-pregnancy weight, prenatal weight, gestational age, parity, mode of delivery, and pregnancy complication, could be obtained and confirmed from the “Maternal Health Manual”. Maternal height and weight were measured and recorded on site by professionally trained pediatric nurses using a unified standard measuring instrument at enrollment, respectively. Subsequently, the weight and height of lactating women were further calculated as body mass index (BMI, kg/m^2^). According to the Chinese adult weight standard [15], BMI was classified into four grades: underweight (<18.5), normal weight (18.5–23.9), overweight (24.0–27.9), and obese (≥28.0).

#### 2.2.3. Collection and Assessment of Dietary Information

FFQ was used to investigate the food frequency and the consumption of lactating women in the past month before enrollment and at the follow-up visit. It was filled out by the investigators at enrollment and the follow-up visit. This FFQ was specially designed by our research group for lactating women and had been validated against a 3-day dietary record [16]. The Spearman correlation coefficients of total fatty acids (TFA), saturated fatty acids (SFAs), monounsaturated fatty acids (MUFAs), and PUFAs of the two measurement methods were 0.48, 0.46, 0.46, and 0.50, respectively.

The raw data of relevant information in FFQ were input into the EpiData database to test the accuracy of the estimation results. Then, the estimation results were input into the dietary evaluation database established based on the “China Food Composition Tables (6th edition) [17]”; the fatty acid data lacking in the database were supplemented by the National Nutrient Database of the United States Department of Agriculture [18] to obtain a relatively perfect food fatty acid database. Finally, the average daily energy and nutrient intake of the research subjects were calculated.

#### 2.2.4. Breast Milk Collection and DHA Level Analysis

Breast milk (10–15 mL) was collected from each subject between 07:30 and 09:00 at enrollment and the follow-up visit. Moreover, breast milk was collected in polypropylene tubes using an electric breast pump, with no preference for one breast. Notably, all the sampling procedures were performed away from direct light, and the divided samples were wrapped with tin foil, stored at −20 °C in a low-temperature refrigerator for one week, and then moved to a −70 °C refrigerator.

Total lipids were extracted from the breast milk samples according to the method suggested by Röse–Gottlieb et al. [19]. Gas chromatography was performed to determine the DHA level in breast milk; the detailed steps of the same have been previously described [20]. Finally, the gas chromatogram of fatty acids (FAs) was obtained (Supplementary Figure S1). The absolute concentrations of FAs were calculated by comparing them with the retention times of the standards and expressed as mg/100 mL to explain the FA contents in 100 mL of breast milk. Then, the relative concentrations of FAs in the breast milk were calculated to explain the compositions of FAs in the breast milk; that is, the weight percentage of TFA (wt.%).

### 2.3. Statistical Analysis

#### 2.3.1. Estimation of Sample Size

Presently, the available literature on the effect of DHA supplement intervention on the composition of breast milk in China is relatively limited. Therefore, we mainly prepared sample estimations based on whether dietary DHA intake met the recommended standard. As the intake of dietary DHA fluctuates greatly in a population, it is not suitable to calculate the sample size by means and standard deviations (mean ± SD), but rather by the corresponding rate. Accordingly, the sample size was calculated using the following formula:

$$n_{1}=n_{2}=\frac{{\left[u_{\alpha }\sqrt{2\pi_{c}\left(1-\pi_{c}\right)}+u_{\beta }\sqrt{\pi_{1}\left(1-\pi_{1}\right)+\pi_{2}\left(1-\pi_{2}\right)}\right]}^{2}}{(\pi_{1}-\pi_{2}{)}^{2}}$$

The effective rates of the control group and the supplement group were estimated to be π_1_ and π_2_, respectively. The merger ratio was π_c_: π_c_ = (π_1_ + π_2_)/2. α: Type I error, take 0.05; β: Type II error, take 0.20.

According to the dietary DHA intake data of lactating women in China at one month postpartum, the standard rate of dietary DHA intake was 7.4%, while the area investigated in the literature was Changchun, whose geographical location and economic level were similar to those of Nanjing [21]. Therefore, if the effective rate of the control group was 7.4%, assuming that the effective rate in the supplement group after DHA supplementation intervention was 20%, then α = 0.05 and β = 0.2 were substituted in the formula to obtain the minimum sample size of *n* = 55 for each group.

#### 2.3.2. Development of Dietary Pattern

To further analyze the DHA intervention effect and establish a dietary pattern for lactating women in the supplement group, 156 types of food in the FFQ were classified into 12 categories according to the “China Food Composition Tables (6th edition) [17]” as follows: cereal and beans other than soybeans, potatoes, vegetables, fruits, livestock meat, poultry meat, fish, shrimp, crab and shellfish, eggs, soybeans and its products, milk and its products, nuts, and cooking oils. The dietary patterns were then derived by the principal component analysis (PCA) with varimax rotation. The Kaiser–Meyer–Olkin test (KMO = 0.556 > 0.5) and Bartlett’s spherical test result (*p* < 0.001) suggested that the data structure was reasonable. Next, according to the scree plot (Supplementary Figure S2), eigenvalue (>1), factor interpretability, and total variance explained, four components were extracted. The eigenvalues of the four patterns were 2.052, 1.965, 1.409, and 1.156, respectively, and explained as 17.10%, 33.47%, 11.74%, and 9.63% of the total variance, respectively.

#### 2.3.3. Data Analysis

The basic information about all lactating women was analyzed, and the dietary energy and nutrient intake of lactating women, and the content of relevant indicators in the breast milk, were statistically described. Categorical variables were expressed as frequency (*n*) and percentage (%), and the differences between groups were compared using the Chi-square test (χ²). Continuous variables were expressed as mean ± SD or median ± interquartile range (median ± IQR). The Kolmogorov–Smirnov test was employed to check whether the data complied with normal distribution, and an independent sample *t*-test (normal distribution) or Mann–Whitney U rank sum test (abnormal distribution) was employed to compare the differences between the two groups simultaneously. A paired *t*-test was performed to compare the levels of relevant indicators in the breast milk of the supplement group and the control group before and after the intervention. In addition, one-way analysis of variance (ANOVA) (normal distribution) or the Kruskal–Wallis H rank sum test (abnormal distribution) was employed to compare the influences of dietary patterns on the intervention effect of the DHA level in breast milk. Tukey’s HSD (normal distribution) or Kruskal–Wallis one-way ANOVA by rank was performed for multiple comparisons. All statistical tests were two-sided, and *p* < 0.05 indicated statistical significance. All statistical analyses of this study were performed using the Statistical Package for the Social Sciences, version (SPSS26.0) (IBM, New York, NY, USA).

## 3. Results

### 3.1. Baseline Information

**Figure 1:**
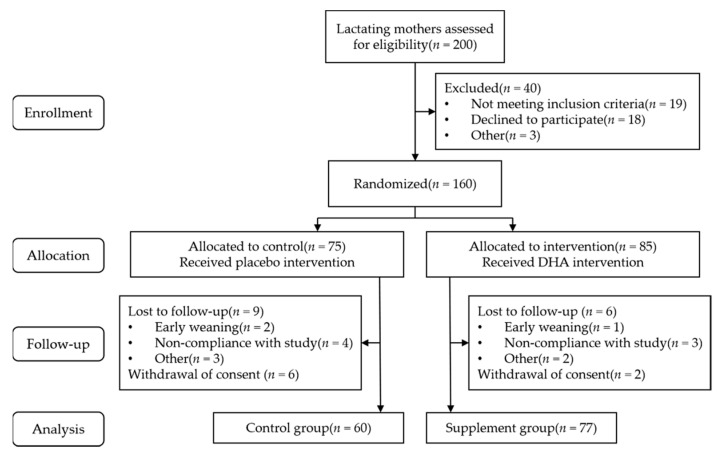
Flow diagram for study subjects.

A total of 160 lactating women were enrolled, and 137 of them completed the study (60 in the control group and 77 in the supplement group). The subject flowchart is shown in Figure 1. The average age, gestational week, and weight gain during pregnancy of the control and supplement groups were 29.8 ± 3.2 and 29.7 ± 4.0 years, 39.0 ± 1.0 and 39.0 ± 1.4 weeks, and 15.0 ± 5.4 and 15.0 ± 5.5 kg, respectively. Most subjects had a university education or higher (63.3% for the control group and 62.3% for the supplement group), and most lactating women were primiparous (66.7% for the control group and 63.6% for the supplement group). The average pre-pregnancy BMI levels of the control and supplement groups were 21.1 ± 2.5 and 21.3 ± 3.8 kg/m^2^, respectively; 76.7% and 70.1% of the subjects in the control and supplement groups, respectively, had normal BMI levels. As shown in Table 1, there were no significant differences in the basic characteristics of the lactating women between the two groups (*p* > 0.05).

### 3.2. Maternal Daily Dietary Energy and Nutrient Intakes between the Two Study Groups at Enrollment and the End of the Trial

In general, there was no statistical difference (*p* > 0.05) in the intake of DHA-related foods (fish, shrimp, crab, and shellfish), regardless of the intervention. At enrollment, the average intakes of fish, shrimp, crab, and shellfish in the control and supplement groups were 55.47 and 47.00 g, respectively; after the intervention, the average intakes in the two groups were 34.46 and 40.00 g, respectively. 

Further analysis of the daily energy and dietary nutrient intake is shown in Table 2. One month before enrollment, the average daily energy intakes of the control and supplement groups were 2335.41 and 2294.49 kcal, respectively. One month before the end of the trial, the average daily energy intakes of the two groups were 2160.21 and 2155.68 kcal, respectively. The energy intakes of the two groups did not exhibit statistically significant differences (*p* > 0.05). According to the macronutrient intake, the protein, fat, carbohydrate intake, and energy provided by the nutrients as percentages of total energy (%E) in the two groups before and after the intervention did not show statistical differences (*p* > 0.05).

Furthermore, one month before the enrollment, the average daily dietary DHA intakes of the control and supplement groups were 48.92 and 40.50 mg, respectively, with no statistically significant differences (*p* > 0.05); one month before the end of the trial, the average daily dietary DHA intakes of the two groups were 31.95 and 33.85 mg, respectively, and did not display statistically significant differences (*p* > 0.05). The intakes of other dietary FAs are shown in Table 2.

### 3.3. Intervention Effect of DHA from Algal Oil on DHA Level in Breast Milk

**Figure 2:**
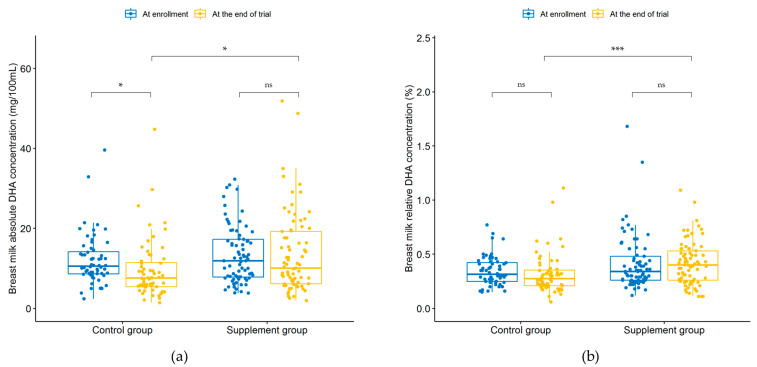
Comparison of the absolute(**a**) and relative (**b**) DHA concentrations in breast milk between the control and supplement groups before and after the intervention. ns: *p* > 0.05, * *p* < 0.05, *** *p* < 0.001.

Table 3 and Table 4 show the absolute (mg/100 mL) and relative concentrations (%) of FAs in the breast milk of lactating women belonging to the control and supplement groups at enrollment and the end of the trial, respectively. Figure 2 presents the differences in DHA content.

In the control group, the absolute concentrations of γ-linolenic acid (GLA), arachidonic acid (ARA), and DHA in the breast milk decreased significantly at the end of the trial (*p* < 0.001, *p* = 0.001 and *p* = 0.037); the relative concentrations of DHA before and after the intervention exhibited no statistical difference (*p* > 0.05). In the supplement group, DHA content in the breast milk before and after the intervention did not show a statistical difference (*p* > 0.05); the relative concentrations of GLA and ARA decreased significantly at the end of the trial (*p* < 0.001). Additionally, the relative concentrations of SFA and ARA/DHA before and after the intervention displayed statistical differences (*p* = 0.013 and *p* = 0.003).

At enrollment, the contents of TFA, butterfat, and various FAs in the breast milk of the two groups had no statistical differences (*p* > 0.05). At the end of the trial, the absolute concentrations of DHA in the breast milk of the control and supplement groups were 7.57 and 10.07 mg/100 mL, respectively, and the relative concentrations were 0.28% and 0.40%, respectively. This finding demonstrates that the absolute and relative concentrations of DHA in the breast milk of the supplement group were significantly higher than those of the control group (*p* = 0.012 and *p* = 0.001). Moreover, the relative concentrations of *n*-6 PUFA, *n*-3 PUFA, and linoleic acid (LA) in the breast milk of the two groups showed statistical differences (*p* < 0.05); *n*-6/*n*-3 PUFA and ARA/DHA in the breast milk of the intervention group were significantly lower than those of the control group (*p* = 0.005 and *p* < 0.001).

### 3.4. Changes in DHA Levels of Breast Milk in the Supplement Group with Respect to Dietary Patterns

In this study, four components that best represented the dietary patterns of lactating mothers and explained 54.84% of the total variance were extracted. The matrix of the factor loadings after the rotation of the four patterns is shown in Supplementary Table S1. For each pattern, the food groups with factor loadings of ≥|0.5| were considered important contributors to the dietary patterns. Pattern 1 mainly included fruits and poultry meat; Pattern 2 was characterized by milk and its products, eggs, fish, shrimp, crab and shellfish, and soybeans and soybean products; Pattern 3 chiefly comprised cereal and beans other than soybeans, potatoes and its products, and nuts; Pattern 4 was high in livestock meat and low in cooking oils.

**Figure 3:**
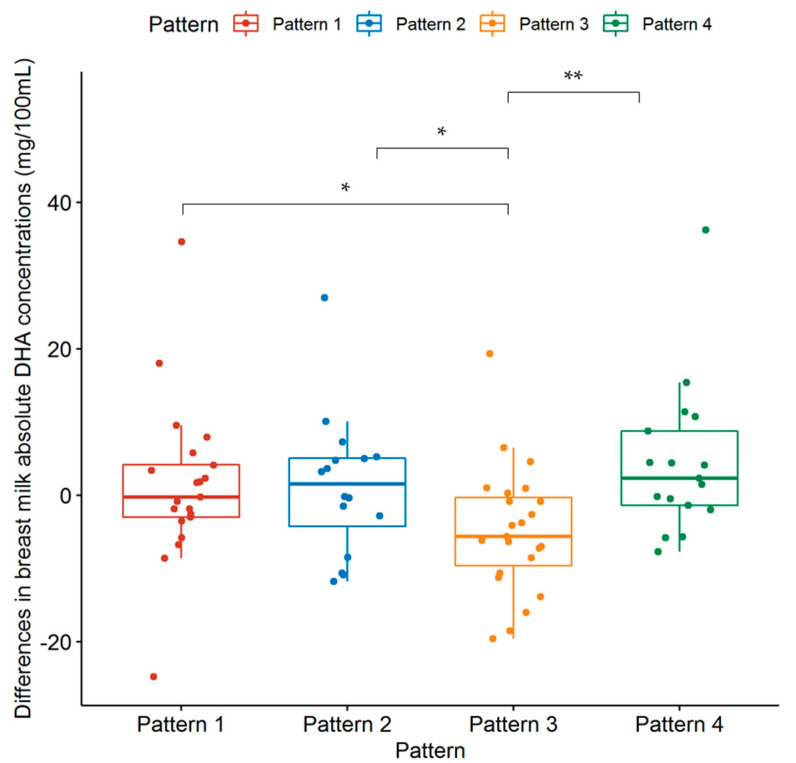
Changes in the absolute DHA concentrations of the breast milk with respect to dietary patterns in the supplement group. * *p* < 0.05, ** *p* < 0.01.

In this study, a total of 21, 16, 23, and 17 subjects from the supplement group were assigned to Pattern 1, Pattern 2, Pattern 3, and Pattern 4, respectively. As shown in Table 5, among the four patterns, the Changes in relative concentrations of DHA in the breast milk of the supplement group before and after the intervention did not exhibit any statistical differences (*p* > 0.05). However, the alterations in the absolute concentrations of DHA in the breast milk among the four dietary patterns showed statistical differences (*p* = 0.013). Further analysis of the differences among the patterns is shown in Figure 3. The DHA level in the breast milk of lactating women from Pattern 3 was significantly lower than from other dietary patterns (*p* < 0.05).

## 4. Discussion

Lactating women were randomly divided into control and supplement groups. After continuously consuming algal DHA oil (200 mg/d) for 8 weeks, the changes in the DHA levels of the breast milk were observed, and the effects of different dietary patterns on the intervention effects were explored. The data showed that, compared with the control group, DHA supplementation increased the DHA content in breast milk. Among the four dietary patterns, the pattern dominated by cereal and beans other than soybeans, potatoes, and nuts exerted a poor intervention effect on DHA.

In 2013, the Chinese Nutrition Society recommended that the appropriate intake of DHA for lactating women was 200 mg/d [22]. However, in this study, whether before or during the intervention, the dietary DHA intake of only a few lactating women reached this recommended level and the average DHA intake was far lower than the recommended amount. This observation agrees with the previous research results on the dietary DHA intake of lactating women in Changchun, China [21]. The U.S. Department of Agriculture has also reported that the seafood intake of many lactating women did not meet the recommended amount, thereby resulting in an inadequate level of DHA intake, approximately 30–70 mg/d [23]. In Addition, for *n*-6/*n*-3 PUFA, the average ratios of lactating women in the months before and during the intervention were 7.84:1 and 7.56:1, respectively, which are slightly higher than the ratio of 4–6:1 recommended by the Chinese Nutrition Society. In fact, the recommended ratios of *n*-6 PUFA and *n*-3 PUFA vary from country to country [2]. The ratio recommended by the United Nations Food and Agriculture Organization is 4–10:1, Canadian scholars: 5–10:1, Japanese scholars: 4:1 [24], and American scholars: 2.3:1 [25]. The slightly higher ratio in this study could be attributed to the higher consumption of vegetable oil by urban residents [26], and the higher LA content of *n*-6 PUFA. Breast milk secretion during lactation requires a high amount of *n*-3 PUFA. Therefore, the dietary sources of *n*-6 PUFA and *n*-3 PUFA in lactating women should reach the appropriate ratio.

In this study, after DHA supplementation, the DHA level in the breast milk of the supplement group was significantly different from that of the control group, which is consistent with previous studies [7,8,27]. Earlier studies have reported that the DHA level in the breast milk of the supplement group showed an upward trend compared with that before the intervention [7], but it was not the case in this study. The DHA level in the breast milk of the control group without DHA supplementation demonstrated a downward trend, which is consistent with previous studies [28]. The reason may be that, in the absence of any intervention, the FA levels in breast milk vary during the entire period of lactation and DHA decreases significantly with lactation progress [29]. Intervention time or dosage may be the restricting factors. In this study, the intervention is insufficient to compensate for the decrease of DHA itself with the progress of lactation. Although ALA can be metabolically converted to DHA in vivo, the activities of carbon chain lengthening enzymes and desaturase necessary for the DHA synthetic pathway are low in infants; hence, the DHA synthetic ability is quite limited [5]. With the development and functional maturity of infants, their ability to synthesize DHA gradually improves, so the DHA content in breast milk decreases accordingly. In this study, it was observed that the ARA levels in the breast milk of both groups decreased significantly owing to a reason similar to that of DHA. ARA is also an important component of the nervous system and is closely related to the intelligence, neural development, and visual acuity of infants [30,31]. Nevertheless, DHA and ARA share the same desaturase and carbon chain lengthening enzyme in the synthetic pathway, which results in a certain competitive relationship [32]. Therefore, the importance of ARA should not be ignored while paying attention to DHA supplementation.

Furthermore, PCA was used to identify four dietary patterns in the lactating women belonging to the intervention group. The statistical analysis led to the conclusion that in Pattern 3, which was dominated by cereal and beans other than soybeans, potatoes, and nuts, the DHA intervention had less obvious effects on the DHA level in breast milk. The FAs in breast milk are not only derived from the endogenous synthesis of mammary glands, but also the intake of maternal blood [33]. Therefore, DHA supplementation, together with other FAs obtained from the diet, maintains the balance of FAs in the mother and meets the demand for milk secretion [34]. Pattern 1, Pattern 2, and Pattern 4 predominantly consisted of animal foods; the maternal dietary intake of *n*-3 PUFA was relatively rich and the changes in DHA levels among the three patterns are relatively close. Although the characteristics of Pattern 2 are fish, shrimp, crab, and shellfish, the main consumption in inland areas involves freshwater fish and shrimp, which are not rich sources of *n*-3 PUFAs [17]. Thus, Pattern 2 did not show a higher change in DHA levels. The diets of lactating women under Pattern 3 mainly consisted of plant-based foods, such as cereal and beans other than soybeans, potatoes, and nuts. The nuts consumed by the lactating women in this study were mostly walnuts, cashews, and chestnuts. The PUFA in these nuts was mainly LA; ALA and DHA contents were quite low, and their daily consumption was relatively small.

The advantage of this study is that it was a randomized controlled experiment with comprehensive data on dietary intake and breast milk. The two groups did not show any difference in basic characteristics and were comparable. Dietary data were obtained via FFQ, which reflected the subjects’ dietary intakes for a prolonged period. Previous studies mostly used fish oil supplements as intervention capsules [35]. These supplements often contain EPA and DHA, whereas the algal oil supplements employed in this study contain only DHA and, hence, accurately reflect the intervention effect of this fatty acid.

This study also exhibits several limitations. The FFQ investigated the food intake within one month. Although the food atlas with sufficient visual reference was used in this study, some recall bias could have been present in the reporting of the dietary data. In addition, foods rich in DHA are fish, shrimp, crab, and shellfish. Our previous study found that this food category is the one with the largest estimation error of edible parts among all food categories, which may also impact the dietary results. Furthermore, the sample size of the control group and the supplement group in this study met our expectations (see Section 2.3.1.). However, we found that at the time of enrollment, almost all of the dietary fatty acid contents in the supplement group were lower than those in the control group. This situation may be improved if the sample size is expanded. Finally, this study did not collect the most intuitive data related to the impact of DHA supplementation on the brain development or cognitive ability of infants. Moreover, the evidence supporting the long-term effects of maternal DHA supplementation on the growth and development of infants is insufficient. Therefore, follow-up studies are required to ascertain the effects of DHA supplementation in lactating women on the long-term brain development or cognitive ability of infants and young children.

## Figures and Tables

**Table 1:** Demographic characteristics of the lactating women in this study.

Characteristics	Control Group	Supplement Group	t/χ²	*p* Value
Age, year (mean ± SD)	29.8 ± 3.2	29.7 ± 4.0	0.193	0.847
Education, *n* (%)			1.333	0.721
High school education or lower	10 (16.7)	11 (14.3)		
Vocational–technical school education	12 (20.0)	18 (23.4)		
University education or higher	38 (63.3)	48 (62.3)		
Gestational age, week	39.0 ± 1.0	39.0 ± 1.4	−0.053	0.958
Parity, *n* (%)			0.136	0.712
1	40 (66.7)	49 (63.6)		
≥2	20 (33.3)	28 (36.4)		
Height, cm	160.5 ± 7.0	162.0 ± 5.0	−0.864	0.388
Pre-pregnancy weight, kg	54.0 ± 9.8	55.0 ± 8.0	−1.273	0.203
Weight at delivery, kg	69.5 ± 12.0	70.0 ± 9.0	−1.390	0.164
Pre-pregnancy BMI, kg/m^2^	21.1 ± 2.5	21.3 ± 3.8	−0.822	0.411
Pre-pregnancy BMI category a, *n* (%)			1.605	0.658
Underweight	6 (10.0)	10 (13.0)		
Normal weight	46 (76.7)	54 (70.1)		
Overweight	7 (11.7)	9 (11.7)		
Obesity	1 (1.7)	4 (5.2)		
Weight gain during pregnancy, kg	15.0 ± 5.4	15.0 ± 5.5	−0.687	0.492
Weight at enrollment, kg	60.0 ± 10.0	61.0 ± 8.75	−1.521	0.128
BMI at enrollment, kg/m^2^	23.3 ± 2.4	23.8 ± 2.8	−1.15	0.252
BMI category at enrollment			1.899	0.387
Underweight	0	0		
Normal weight	36 (60.0)	43 (55.8)		
Overweight	23 (38.3)	29 (37.7)		
Obesity	1 (1.7)	5 (6.5)		

Data are expressed as median ± IQR, or number and percentage (*n* (%)) for categorical variables unless otherwise specified. *p* values were assessed using the Mann–Whitney U rank sum test for non-normally distributed continuous variables or the χ² test for categorical variables.

**Table 2:** Maternal dietary intakes of energy and nutrients between the control and supplement groups at the time of enrollment and the end of the trial.

Intakes	At Enrollment			At the End of the Trial		
	Control Group	Supplement Group	*p* Value	Control Group	Supplement Group	*p* Value
Energy, kcal/d	2335.41 ± 715.35	2294.49 ± 583.43	0.374 ^Δ^	2160.21 ± 824.95	2155.68 ± 579.99	0.484 ^Δ^
Protein, g/d	101.99 ± 35.56	96.18 ± 44.33	0.338	86.15 ± 41.01	87.48 ± 33.79	0.890
Protein, %E	17.23 ± 4.38	17.42 ± 4.84	0.632 ^Δ^	15.87 ± 3.53	16.71 ± 3.93	0.397 ^Δ^
Fat, g/d	89.11 ± 28.41	85.86 ± 26.69	0.314	78.66 ± 34.78	78.42 ± 21.77	0.549
Fat, %E	33.53 ± 8.86	33.75 ± 7.98	0.603	33.11 ± 10.08	33.09 ± 7.53	0.535
Carbohydrates, g/d	290.37 ± 110.80	282.82 ± 108.31	0.799 ^Δ^	273.86 ± 121.95	267.50 ± 112.06	0.652
Carbohydrates, %E	48.89 ± 11.50	49.52 ± 10.71	0.478 ^Δ^	50.90 ± 10.86	51.40 ± 9.88	0.709 ^Δ^
Total fatty acids, g/d	78.86 ± 19.96	74.49 ± 20.77	0.270	67.74 ± 30.97	68.71 ± 22.14	0.758
SFA, g/d	20.73 ± 7.07	20.76 ± 8.74	0.528 ^Δ^	17.25 ± 9.93	17.75 ± 8.74	0.972
SFA, %E	8.06 ± 3.08	8.03 ± 2.69	0.986	7.71 ± 3.00	7.83 ± 2.87	0.853 ^Δ^
MUFA, g/d	33.84 ± 12.19	32.39 ± 11.25	0.304	30.45 ± 14.37	30.62 ± 9.81	0.330 ^Δ^
PUFA, g/d	23.26 ± 6.95	22.02 ± 7.12	0.223	20.77 ± 7.95	20.87 ± 9.80	0.591
PA, g/d	11.59 ± 7.42	10.42 ± 7.22	0.265	9.43 ± 9.16	8.43 ± 7.54	0.549
SA, g/d	4.89 ± 3.14	4.37 ± 3.32	0.524	3.57 ± 3.62	3.70 ± 2.95	0.938
POA, g/d	1.25 ± 1.03	1.15 ± 1.08	0.410	0.91 ± 0.92	0.88 ± 0.86	0.818
OA, g/d	22.47 ± 14.01	18.64 ± 12.96	0.187	17.04 ± 19.48	15.76 ± 14.10	0.541
LA, g/d	17.61 ± 10.81	14.98 ± 9.60	0.072	13.24 ± 9.65	13.44 ± 9.83	0.448
ALA, g/d	2.43 ± 3.66	2.13 ± 1.37	0.087	2.05 ± 2.25	1.84 ± 1.48	0.371
ARA, g/d	0.23 ± 0.36	0.22 ± 0.26	0.410	0.17 ± 0.21	0.17 ± 0.23	0.835
EPA, g/d	36.19 ± 59.28	32.04 ± 72.04	0.371	17.93 ± 46.02	18.92 ± 59.34	0.619
DHA, g/d	48.92 ± 51.73	40.5 ± 50.69	0.211	31.95 ± 30.65	33.85 ± 43.37	0.607
*n*-3 PUFA, g/d	2.57 ± 3.78	2.20 ± 1.37	0.077	2.14 ± 2.30	1.88 ± 1.59	0.367
*n*-3 PUFA, %E	0.94 ± 1.07	0.85 ± 0.52	0.077	0.96 ± 0.87	0.88 ± 0.52	0.333
*n*-6 PUFA, g/d	17.74 ± 10.87	15.18 ± 9.83	0.072	13.56 ± 9.93	13.51 ± 10.00	0.415
*n*-6 PUFA, %E	6.75 ± 3.46	6.26 ± 3.37	0.066	5.64 ± 4.83	5.68 ± 3.58	0.344
*n*-6/*n*-3 PUFA	6.66 ± 5.67	6.88 ± 5.00	0.349	6.45 ± 4.81	6.80 ± 5.19	0.872

^Δ^*p* values were assessed using independent samples *t*-test for normally distributed continuous variables. %E, the percentage of energy provided by the nutrient as a percentage of total energy. SFA, saturated fatty acid; MUFA, monounsaturated fatty acid; PUFA, polyunsaturated fatty acid; PA, palmitic acid); SA, stearic acid; POA, palmitoleic acid; OA, oleic acid; LA, linoleic acid; ALA, α-linolenic acid; ARA, arachidonic acid; DHA, docosahexaenoic acid; EPA, eicosapentaenoic acid.

**Table 3:** The absolute concentrations of fatty acids in breast milk between the control and supplement groups at the time of enrollment and the end of the trial.

Fatty Acid, mg/100 mL	Control Group		Supplement Group		*p* Value ^a^	*p* Value ^b^	*p* Value ^c^	*p* Value ^d^
	At Enrollment	At the End of the Trial	At Enrollment	At the End of the Trial				
Milk fat, g/100 mL	3.86 ± 1.61	3.14 ± 2.03	3.70 ± 2.15	3.47 ± 2.30	0.483	0.716	0.128	0.688
Total fatty acids	3350.65 ± 1368.64	2766.48 ± 1581.62	3139.61 ± 1813.13	3018.65 ± 2039.50	0.306	0.712	0.122	0.895
SFA	1118.94 ± 396.45	962.79 ± 606.38	1039.70 ± 631.60	980.27 ± 617.43	0.140	0.661	0.116	0.810
MUFA	1212.41 ± 599.12	1100.84 ± 741.44	1243.36 ± 755.57	1134.22 ± 819.33	0.828	0.535	0.080	0.626
PUFA	884.47 ± 343.77	746.51 ± 503.29	813.39 ± 373.08	786.61 ± 520.33	0.288	0.938	0.327	0.920
*n*-6 PUFA	807.80 ± 319.15	705.87 ± 463.12	733.88 ± 321.12	678.83 ± 474.72	0.241	0.805	0.430	0.895
*n*-3 PUFA	64.22 ± 44.33	60.48 ± 41.55	69.08 ± 48.19	69.73 ± 53.42	0.398	0.146	0.430	0.806
*n*-6/*n*-3	11.92 ± 6.67	11.79 ± 8.04	10.72 ± 5.40	9.92 ± 6.01	0.136	0.005	0.534	0.059
LA	731.60 ± 290.04	657.96 ± 400.72	664.96 ± 278.77	634.92 ± 445.66	0.247	0.768	0.467	0.937
GLA	6.16 ± 3.81	3.87 ± 2.91	5.10 ± 4.24	3.92 ± 2.80	0.243	0.811	<0.001	0.093
ALA	46.71 ± 37.85	46.81 ± 42.16	50.32 ± 39.27	51.31 ± 47.02	0.443	0.233	0.697	0.788
ARA	21.68 ± 9.59	15.54 ± 9.97	21.54 ± 15.15	16.61 ± 12.74	0.771	0.671	0.001	0.031
EPA	1.33 ± 1.30	1.26 ± 1.30	1.46 ± 1.87	1.53 ± 2.05	0.828	0.335	0.928	0.244
DHA	10.55 ± 5.84	7.57 ± 6.30	11.88 ± 9.59	10.07 ± 13.16	0.531	0.012	0.037	0.873
ARA/DHA	2.01 ± 0.85	2.06 ± 1.30	2.00 ± 0.90	1.47 ± 1.06	0.742	<0.001	0.328	0.003

SFA, saturated fatty acid; MUFA, monounsaturated fatty acid; PUFA, polyunsaturated fatty acid; LA, linoleic acid; GLA, γ-linolenic acid; ALA, α-linolenic acid; ARA, arachidonic acid; DHA, docosahexaenoic acid; EPA, eicosapentaenoic acid. ^a^ At enrollment: control group vs. supplement group; ^b^ At the end of the trial: control group vs. supplement group; ^c^ control group: at enrollment vs. at the end of the trial; ^d^ supplement group: At enrollment vs. At the end of the trial.

**Table 4:** The relative concentrations of fatty acids in breast milk between the control and supplement groups at the time of enrollment and the end of the trial.

Fatty Acid, %	Control Group		Supplement Group		*p* Value ^a^	*p* Value ^b^	*p* Value ^c^	*p* Value ^d^
	At Enrollment	At the End of the Trial	At Enrollment	At the End of the Trial				
SFA	34.99 ± 5.62	34.88 ± 4.52	33.85 ± 4.45	34.76 ± 5.56	0.063 ^Δ^	0.910	0.647	0.013
MUFA	38.25 ± 5.40	37.79 ± 6.77	39.58 ± 5.00	38.43 ± 5.15	0.072 ^Δ^	0.328 ^Δ^	0.630	0.062
PUFA	26.44 ± 6.82	26.84 ± 5.06	26.72 ± 5.37	25.74 ± 5.84	0.973 ^Δ^	0.124 ^Δ^	0.442	0.290
*n*-6 PUFA	24.49 ± 6.36	24.67 ± 5.25	24.47 ± 5.22	23.08 ± 5.07	0.976	0.044 ^Δ^	0.558	0.142
*n*-3 PUFA	1.87 ± 0.93	2.12 ± 1.23	2.15 ± 0.98	2.41 ± 1.13	0.062	0.036	0.285	0.152
LA	22.41 ± 6.00	23.30 ± 5.42	22.10 ± 4.84	21.16 ± 5.13	0.879	0.043 ^Δ^	0.285	0.476
GLA	0.18 ± 0.11	0.14 ± 0.08	0.16 ± 0.08	0.13 ± 0.07	0.286 ^Δ^	0.499	<0.001	<0.001
ALA	1.5 ± 0.78	1.63 ± 1.05	1.64 ± 0.75	1.83 ± 1.18	0.151	0.101	0.166	0.144
ARA	0.65 ± 0.23	0.56 ± 0.17	0.70 ± 0.23	0.59 ± 0.20	0.119 ^Δ^	0.642	<0.001	<0.001
EPA	0.04 ± 0.03	0.05 ± 0.04	0.04 ± 0.03	0.06 ± 0.04	0.706	0.422	0.316	0.284
DHA	0.32 ± 0.18	0.28 ± 0.15	0.34 ± 0.23	0.40 ± 0.29	0.248	0.001	0.277	0.858

^Δ^ *p* values were assessed using independent samples *t*-test for normally distributed continuous variables. SFA, saturated fatty acid; MUFA, monounsaturated fatty acid; PUFA, polyunsaturated fatty acid; LA, linoleic acid; GLA, γ-linolenic acid; ALA, α-linolenic acid; ARA, arachidonic acid; DHA, docosahexaenoic acid; EPA, eicosapentaenoic acid. ^a^ At enrollment: control group vs. supplement group; ^b^ at the end of the trial: control group vs. supplement group; ^c^ control group: at enrollment vs. at the end of the trial; ^d^ supplement group: at enrollment vs. at the end of the trial.

**Table 5:** Changes in DHA concentrations of breast milk with respect to dietary patterns in the supplement group.

DHA	Pattern 1 (*n* = 21)	Pattern 2 (*n* = 16)	Pattern 3 (*n* = 23)	Pattern 4 (*n* = 17)	*p* Value
Absolute concentration, mg/100 mL	−0.23 ± 8.22	1.52 ± 12.25	−5.61 ± 10.91	2.29 ± 11.46	0.013
Relative concentration, %	0.05 ± 0.20	0 ± 0.39	−0.08 ± 0.24	0.07 ± 0.20	0.267 ^Δ^

^Δ^ *p* values were assessed using the analysis of variance for normally distributed continuous variables. *p* values were assessed using the Kruskal–Wallis test for non-normally distributed continuous variables.

## Data Availability

The data presented in this study are available upon request from the corresponding author.

## Supplementary Materials

The following supporting information can be downloaded at: https://www.mdpi.com/article/10.3390/nu14163410/s1, Figure S1: Gas chromatogram of fatty acids in breast milk. Figure S2: Scree plot of eigenvalues for the 12 kinds of food based on principal component analysis with varimax rotation; Table S1: Rotated component matrix among the different patterns.
