# Supplementary material for: Impact of DHA from Algal Oil on the Breast Milk DHA Levels of Lactating Women: A Randomized Controlled Trial in China
Source: Nutrients. 2022 Aug 19;14(16):3410. doi: 10.3390/nu14163410 (PMC9415549; doi:10.3390/nu14163410)
Supplement: Supplementary file 1 [file nutrients-14-03410-s001.zip › nutrients-1865711-supplementary.pdf]

Supplementary information

Table S1. Rotated component matrix among the different patterns.

Food group	Score coefficient			
	Pattern 1	Pattern 2	Pattern 3	Pattern 4
Fruits	**0.831**	0.207	0.091	-0.188
poultry meat	**0.779**	-0.067	-0.024	0.309
Milk and its products	0.116	**0.787**	-0.052	-0.013
Eggs	-0.319	**0.617**	-0.161	0.016
Fish, shrimp and shellfish	0.158	**0.581**	-0.125	-0.069
Soybeans and its products	0.098	**0.531**	0.230	0.415
Cereals and beans other than soybeans	-0.296	-0.171	**0.749**	-0.177
Potatoes	0.213	0.006	**0.631**	-0.119
Nuts	0.453	0.089	**0.592**	-0.100
Vegetables	-0.035	-0.180	0.432	0.214
Livestock meat	-0.073	0.080	-0.058	**0.748**
Cooking oils	-0.090	0.074	0.087	**-0.672**

The food groups with factor loadings of ≥|0.5| are for bold in the table

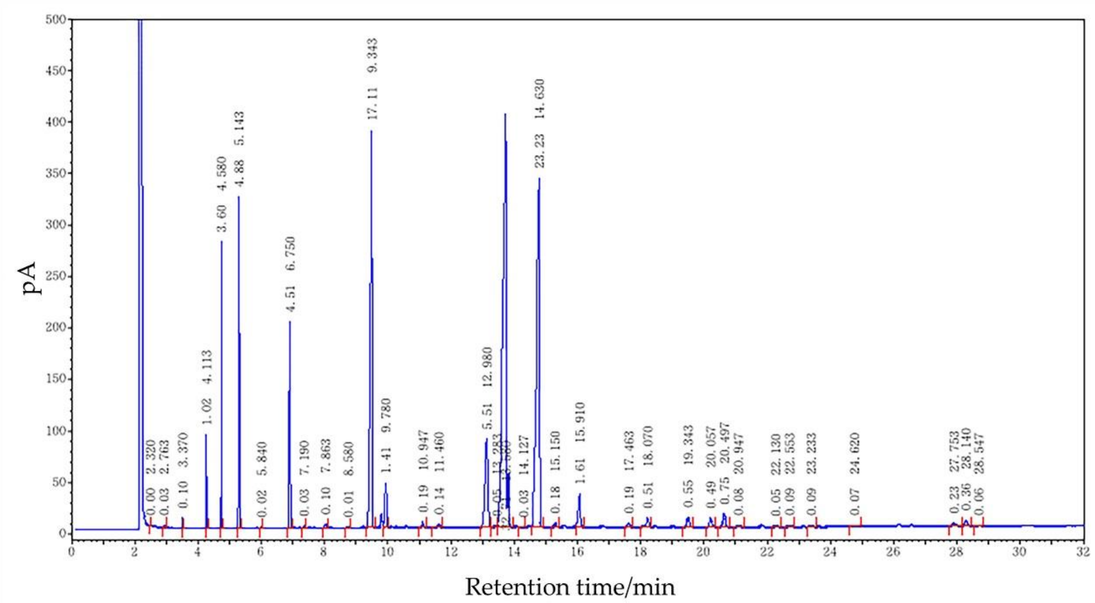

Figure S1. Gas chromatogram of fatty acids in breast milk

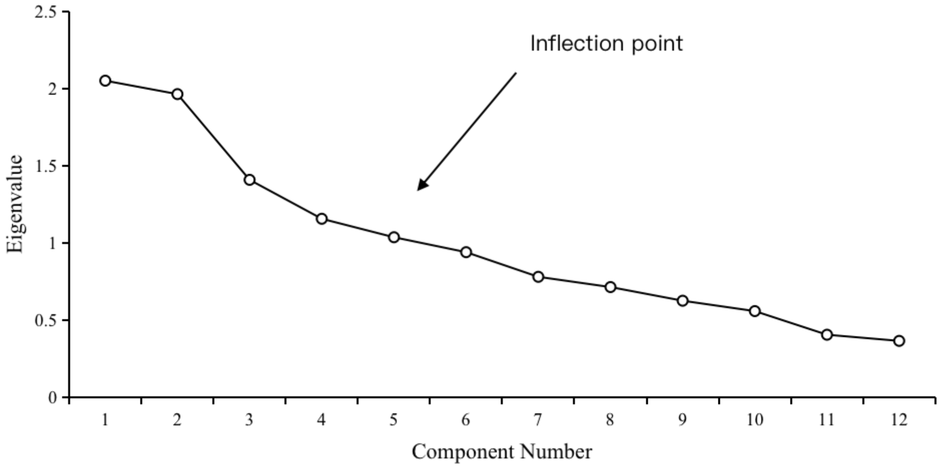

Figure S2. Scree plot of eigenvalues for the 12 kinds of food based on principal component analysis with varimax rotation.
Components with an eigenvalue >1 were retained.
